# Type 2 Diabetes Status, Diabetes Complication Severity Index Scores, and Their Relationship With COVID-19 Severity: A Retrospective Cohort Study of Hospitalized Patients in a Southwest Virginia Health System

**DOI:** 10.7759/cureus.53524

**Published:** 2024-02-03

**Authors:** Jesse G Bendetson, Anthony W Baffoe-Bonnie

**Affiliations:** 1 Department of Medicine, Virginia Tech Carilion School of Medicine, Roanoke, USA; 2 Section of Infectious Diseases, Department of Medicine, Virginia Tech Carilion School of Medicine, Roanoke, USA

**Keywords:** retrospective cohort study, diabetes complications severity index, type 2 diabetes, in-hospital outcomes, covid-19

## Abstract

Background

Studies have shown that patients with type 2 diabetes mellitus (T2DM) tend to have poorer outcomes associated with COVID-19, including increased rates of hospitalization, ICU admission, need for ventilatory support, and mortality.

Methods

We performed a retrospective cohort study that included all non-pregnant adult patients who were hospitalized as a result of COVID-19 in a Southwest Virginia health system between March 18, 2020, and August 31, 2022. T2DM status was treated as a binary variable. T2DM severity was assessed using the Diabetes Complications Severity Index (DCSI). Multivariate logistic regression was used to assess the relationship between T2DM status and COVID-19 severity outcomes. Multivariate logistic regression was also used to assess the relationship between DCSI score and COVID-19 severity outcomes among patients with an established diagnosis of T2DM at the time of COVID-19 hospital admission.

Results

Patients with T2DM had 1.27 times the odds of experiencing a poor COVID-19 clinical outcome (95% CI: 1.13, 1.43) and 1.35 times the odds of in-hospital mortality (95% CI: 1.14, 1.59) compared to patients without diabetes. Among patients with T2DM, increasing DCSI score was significantly associated with increased odds of experiencing a poor COVID-19 clinical outcome and in-hospital mortality.

Conclusions

Diabetic patients in our sample were at increased odds of experiencing poor COVID-19 clinical outcomes and in-hospital mortality compared to individuals without diabetes. Amongst patients with T2DM, increasing DCSI score was associated with worse COVID-19 outcomes. Clinical decision support tools may be able to utilize DCSI scores as an indicator of COVID-19 severity risk to facilitate decisions regarding treatment aggressiveness and resource allocation.

## Introduction

Coronavirus disease 2019 (COVID-19) was declared a public health emergency of international significance on January 30, 2020 [1]. As of December 25, 2023, there have been more than 772 million cases of COVID-19 and over 6.9 million deaths worldwide, including more than 103 million cases and over 1.1 million deaths in the United States alone [2]. Although COVID-19 was originally thought to primarily affect the respiratory system, we have since learned that COVID-19 patients experience adverse effects across numerous organ systems [3,4].

COVID-19 can present as an asymptomatic condition; a mild illness with symptoms that may include fever, cough, sore throat, malaise, headache, muscle pain, nausea, vomiting, diarrhea, and loss of taste and smell; or a moderate illness where evidence of lower respiratory disease exists but with pulse oximetry ≥94% on room air. Patients may also experience severe illness with pulse oximetry <94% on room air with most requiring hospitalization, or critical illness involving respiratory or multi-organ failure with patients requiring intensive care unit (ICU) admission and/or ventilatory support [5].

Diabetes mellitus, an endocrine disorder characterized by dysregulation of glucose metabolism, has an enormous impact on individual and public health in the United States. As of 2020, 34.2 million Americans had diabetes, accounting for 10.5% of the U.S. population [6]. Diabetes is the seventh leading cause of mortality in the United States, with 87,647 diabetes-related deaths recorded in 2019 [7]. Additionally, the prevalence of diabetes is increasing at an alarming rate; 1.5 million new cases of diabetes were diagnosed in the U.S. in 2018, representing an incidence rate of 6.9 new cases per 1,000 person-years [6].

Multiple studies have observed that patients with diabetes who develop COVID-19 are at increased risk of hospitalization, longer inpatient stays, ICU admission, and death than non-diabetics [8-11]. Among diabetics, elevated measures of glycemic control including hemoglobin A1c and fasting plasma glucose have been suggested, albeit not consistently, to be predictive of poorer COVID-19 outcomes [12,13].

The present study was designed to add to the mounting literature describing the adverse impact of diabetes status and severity on clinical outcomes associated with COVID-19. We hypothesized that patients with a preexisting diagnosis of type 2 diabetes mellitus (T2DM) who were hospitalized as a result of COVID-19 would be at increased risk of poor clinical outcomes including ICU admission, need for ventilatory support, and death compared to patients without a history of diabetes. We further hypothesized that among patients with T2DM who were hospitalized for COVID-19, those with more severe diabetes would experience poorer clinical outcomes characterized by higher rates of ICU admission, ventilatory support, and death than those with less severe diabetic disease. Our study is unique in that it employs a diabetes severity index that is reproducible across health systems and can be seamlessly integrated into an electronic medical record clinical decision support program to augment clinical care in this high-risk population.

Partial data from this study were presented at the IDWeek 2022 conference in October 2022 as a poster, which was also published as an abstract in the December 2022 supplemental issue of Open Forum Infectious Diseases (https://doi.org/10.1093/ofid/ofac492.903).

## Materials and methods

Study design and participants

We performed a retrospective cohort study that included all non-pregnant adult patients who were hospitalized as a result of COVID-19 between March 18, 2020, and August 31, 2022, at Carilion Clinic, a not-for-profit health system in Southwest Virginia comprised of six acute care hospitals. Patients were eligible if they met the following criteria: (1) 18 years of age or older; (2) hospitalized as a result of COVID-19; (3) not pregnant at the time of COVID-19 hospitalization; and (4) no past diagnosis of type 1 diabetes mellitus. Pregnant patients were excluded to ensure that the well-documented effects of pregnancy on glucose metabolism [14,15] would not introduce bias into our results. There were 6,983 patients who met our eligibility criteria.

Data collection

All relevant data were abstracted from the health system’s electronic medical record (EMR). Variables including T2DM status, sex, race/ethnicity, current smoking status, BMI, COVID-19 vaccination record, and type of hospital unit during admission were abstracted directly from the EMR. Vaccination status at the time of hospitalization and COVID-19 clinical severity were calculated for each patient based on data abstracted from the EMR.

Definitions

The primary outcome for all the analyses we conducted was COVID-19 severity during the hospital encounter. We broke this outcome down two ways, treating it both as a binary “poor COVID-19 clinical outcome” variable and as a binary “COVID-19 in-hospital mortality” variable. The binary poor COVID-19 clinical outcome variable was categorized as follows: (1) patient was admitted to the ICU, required ventilatory support, and/or died in the hospital; and (2) patient was not admitted to the ICU, did not require ventilatory support, and did not die in the hospital. The binary COVID-19 in-hospital mortality variable was categorized as follows: (1) patient died in the hospital; and (2) patient did not die in the hospital.

We used two predictor variables in our analyses. The first predictor variable we used was T2DM status, treated as a binary variable: (1) the patient had an established diagnosis of T2DM documented in the medical record at the time of COVID-19 hospitalization; and (2) the patient did not have an established diagnosis of T2DM documented in the medical record at the time of COVID-19 hospitalization. The second predictor variable we used was the Diabetes Complications Severity Index (DCSI), treated as an ordinal variable. The DCSI is a validated scale based on International Classification of Diseases 10th Revision (ICD-10) codes that provides a 13-point diabetes severity summary score based on seven discrete categories of diabetic complications, as displayed in Table 1 [16-18]. (See the Appendix (Supplementary Methods) for additional details.)

**Table 1:** DCSI Categories of Diabetic Complications

DCSI Category
1. Retinopathy (0-2)
2. Nephropathy (0-2)
3. Neuropathy (0-1)
4. Cerebrovascular disease (0-2)
5. Cardiovascular disease (0-2)
6. Peripheral vascular disease (0-2)
7. Metabolic disease (0-2)

We included age, sex, race/ethnicity, current smoking status, BMI, and vaccination status as covariates in our analyses. Vaccination status was broken down into three categories based on timing of vaccination and date of hospital admission [19]: (1) not vaccinated - patient did not receive any COVID-19 vaccines or received a single dose of a COVID-19 vaccine less than 14 days prior to hospital admission; (2) fully vaccinated, up-to-date - hospital admission occurred: between 14 and 180 days after receiving one dose of the Johnson & Johnson vaccine; between 14 and 180 days after receiving a second dose of an mRNA vaccine; between 14 and 365 days after receiving a third or greater dose of an mRNA vaccine; between 14 and 365 days after receiving one dose of the Johnson & Johnson vaccine preceded by one dose of an mRNA vaccine; and/or between 14 and 365 days after receiving one dose of an mRNA vaccine preceded by one dose of the Johnson & Johnson vaccine; and (3) partially vaccinated, not up-to-date - patient received one or more COVID-19 vaccine dose(s) 14 days or more prior to hospital admission but did not meet the criteria for full vaccination.

Statistical analysis

Analysis of missing data for both categorical and continuous variables revealed that data were missing completely at random (MCAR). As a result, we conducted a complete case analysis and excluded 1,479 cases with missing data on one or more variables, yielding a final sample size of n = 5,504. (See the Appendix (Supplementary Methods) for additional details.)

Multivariate logistic regression was used to assess the relationship between T2DM status and poor COVID-19 clinical outcome and the relationship between T2DM status and COVID-19 in-hospital mortality. Multivariate logistic regression was also used to assess the relationship between DCSI score and poor COVID-19 clinical outcome and between DCSI score and COVID-19 in-hospital mortality among patients with an established diagnosis of T2DM documented in the medical record at the time of COVID-19 hospitalization. All multivariate models included age, sex, race/ethnicity, current smoking status, BMI, and vaccination status as covariates, all of which represent clinically relevant factors for patient outcomes amongst patients hospitalized with COVID-19.

## Results

**Figure 1:**
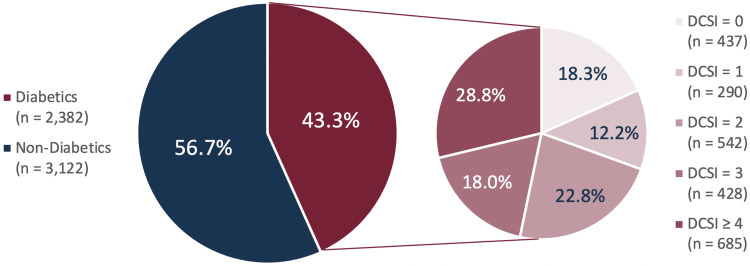
Distributions of T2DM Status Among the Total Study Population (n = 5,504) and DCSI Scores Among Diabetics (n = 2,382)

The characteristics of our study sample, including demographics, other covariates, and COVID-19 severity outcomes, are displayed in Table 2, which presents attributes of the entire sample and among groups that have been stratified according to T2DM status. The distribution of T2DM status among the entire study population and the distribution of DCSI scores among diabetics are displayed in Figure 1.

**Table 2:** Clinical, Demographic, and Outcome Features of Patients Hospitalized With COVID-19

	Total (n = 5,504)	Diabetics (n = 2,382)	Non-diabetics (n = 3,122)	
Variable	n (%)	n (%)	n (%)	χ^2 ^p-value
Sex				0.0003
Female	2,797 (50.8%)	1,144 (48.0%)	1,653 (53.0%)	
Male	2,707 (49.2%)	1,238 (52.0%)	1,469 (47.0%)	
Race/Ethnicity				0.0033
African American	597 (10.9%)	298 (12.5%)	299 (9.6%)	
Caucasian	4,719 (85.7%)	2,017 (84.7%)	2,702 (86.6%)	
Hispanic	114 (2.1%)	40 (1.7%)	74 (2.4%)	
Other/Multiracial	74 (1.3%)	27 (1.1%)	47 (1.5%)	
Age				<0.0001
18-29	228 (4.1%)	13 (0.6%)	215 (6.9%)	
30-39	280 (5.1%)	62 (2.6%)	218 (7.0%)	
40-49	447 (8.1%)	155 (6.5%)	292 (9.4%)	
50-59	914 (16.6%)	420 (17.6%)	494 (15.8%)	
60-69	1,252 (22.8%)	615 (25.8%)	637 (20.4%)	
70-79	1,353 (24.6%)	696 (29.2%)	657 (21.0%)	
80+	1,030 (18.7%)	421 (17.7%)	609 (19.5%)	
Smoking Status				0.0136
Current Smoker	1,890 (34.3%)	861 (36.1%)	1,029 (33.0%)	
Non-smoker	3,614 (65.7%)	1,521 (63.9%)	2,093 (67.0%)	
Body Mass Index				<0.0001
Underweight (BMI <18.5)	165 (3.0%)	27 (1.1%)	138 (4.4%)	
Normal Weight (BMI 18.5-24.9)	1,092 (19.8%)	351 (14.7%)	741 (23.7%)	
Overweight (BMI 25.0-29.9)	1,490 (27.1%)	605 (25.4%)	885 (28.4%)	
Obese (BMI 30.0-34.9)	1,143 (20.8%)	546 (22.9%)	597 (19.1%)	
Morbidly Obese (BMI ≥35.0)	1,614 (29.3%)	853 (35.8%)	761 (24.4%)	
Vaccination Status				0.0009
Not Vaccinated	4,152 (75.4%)	1,743 (73.2%)	2,409 (77.2%)	
Partially Vaccinated	742 (13.5%)	365 (15.3%)	377 (12.1%)	
Fully Vaccinated	610 (11.1%)	274 (11.5%)	336 (10.8%)	
DCSI Score				--
0		437 (18.4%)		
1		290 (12.2%)		
2		542 (22.8%)		
3		428 (18.0%)		
4+		685 (28.8%)		
Poor COVID-19 Clinical Outcome	1,977 (35.9%)	960 (40.3%)	1,017 (32.6%)	<0.0001
COVID-19 In-Hospital Mortality	714 (13.0%)	370 (15.5%)	344 (11.0%)	<0.0001

Our study population was split relatively evenly across sex categories, with 50.8% female patients and 49.2% male patients. The large majority of patients (85.7%) identified as Caucasian, with 10.9% identifying as African American and 2.1% identifying as Hispanic. Our sample skewed heavily toward the older age categories with less than 10% of patients under the age of 40. Additionally, 11.1% of patients were fully vaccinated or up-to-date at the time they were admitted to the hospital, 13.5% were partially vaccinated, and 75.4% were not vaccinated. Turning now to COVID-19 severity, 35.9% of patients met our criteria for poor COVID-19 clinical outcome (i.e., were admitted to ICU, required ventilatory support, and/or died in the hospital); this included 40.3% of individuals with T2DM and 32.6% of non-diabetics. Finally, 13.0% of our sample died in the hospital, including 15.5% of patients with T2DM and 11.0% of non-diabetics.

Diabetics compared to non-diabetics

**Figure 2:**
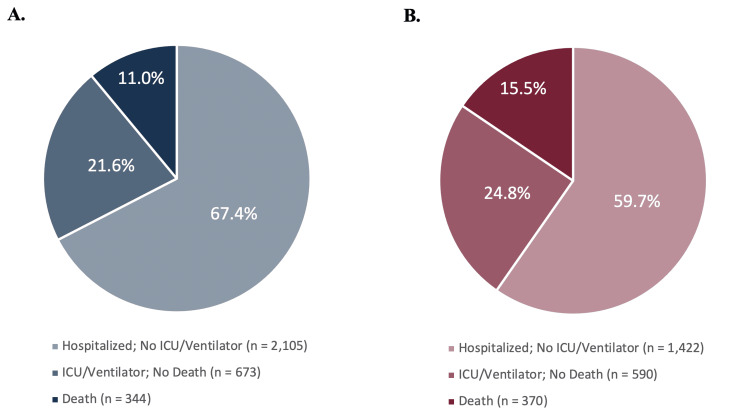
Distribution of COVID-19 Outcomes Amongst Non-diabetics and Diabetics A. Non-diabetics (n = 3,122); B. Diabetics (n = 2,382)

Figure 2 provides a visual representation of the distribution of COVID-19 severity outcomes among patients in our sample, stratified by T2DM status. Table 3 presents the results of our multivariate logistic regression analyses examining the relationships between T2DM status and poor COVID-19 clinical outcome and between T2DM status and COVID-19 in-hospital mortality. After adjusting for potential confounding by age, sex, race/ethnicity, current smoking status, BMI, and vaccination status, diabetic patients in our sample had 1.27 times the odds of experiencing a poor COVID-19 clinical outcome compared to individuals without T2DM (95% CI: 1.13-1.43). Additionally, patients with diabetes were at 1.35 times the odds of in-hospital mortality compared to non-diabetic individuals (95% CI: 1.14, 1.59).

**Table 3:** Multivariate Logistic Regression – Diabetes Status and COVID-19 Severity Outcomes (n = 5,504) * Statistically significant at α = 0.05.

COVID-19 Severity Indicator	Odds Ratio	95% Confidence Interval
Progression to ICU admission, ventilatory support, and/or death	1.27*	1.13, 1.43
In-hospital mortality	1.35*	1.21, 1.58

DCSI scores among diabetics

**Figure 3:**
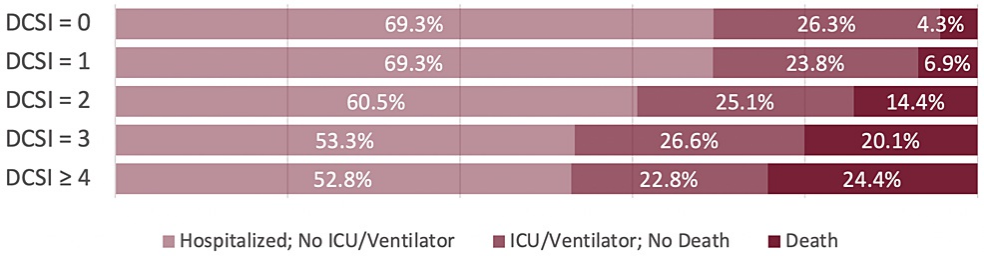
COVID-19 Outcomes Among Diabetics by DCSI Score (n = 2,382)

Figure 3 displays the distribution of COVID-19 severity outcomes among diabetics stratified by DCSI scores. Table 4 displays the results of our multivariate logistic regression of poor COVID-19 clinical outcome on DCSI score among patients with T2DM. Table 5 presents the results of our multivariate logistic regression of COVID-19 in-hospital mortality on DCSI score among patients with T2DM. After controlling for potential confounding by age, sex, race/ethnicity, current smoking status, BMI, and vaccination status, increasing DCSI score was associated with increased odds of poor COVID-19 clinical outcomes and COVID-19 in-hospital mortality. While the comparisons between a DCSI score of 0 and a DCSI score of 1 did not achieve statistical significance at α = 0.05, patients with DCSI score of 2, 3, and 4+ had significantly increased odds of experiencing a poor COVID-19 clinical outcome and COVID-19 in-hospital mortality compared to individuals with a DCSI score of 0.

**Table 4:** Multivariate Logistic Regression – DCSI Score and Poor COVID-19 Clinical Outcome Among Diabetics (n = 2,382) * Statistically significant at α = 0.05.

DCSI Score	Odds Ratio	95% Confidence Interval
0	--	--
1	1.03	0.74, 1.43
2	1.54*	1.16, 2.04
3	2.19*	1.63, 2.95
4+	2.29*	1.74, 3.02

**Table 5:** Multivariate Logistic Regression – DCSI Score and COVID-19 In-Hospital Mortality Among Diabetics (n = 2,382) * Statistically significant at α = 0.05.

DCSI Score	Odds Ratio	95% Confidence Interval
0	--	--
1	1.52	0.79, 2.93
2	3.39*	1.98, 5.80
3	5.03*	2.94, 8.62
4+	6.61*	3.94, 11.07

## Discussion

Our data were consistent in providing evidence for a relationship between T2DM and worsening COVID-19 severity. Specifically, patients with T2DM had a statistically significant increase in the odds of experiencing a poor COVID-19 clinical outcome (OR = 1.27, 95% CI: 1.13, 1.43) and in-hospital mortality (OR = 1.35, 95% CI: 1.14, 1.59) when compared with non-diabetic patients. These results are in line with the many studies that have been performed to present which have found poorer clinical outcomes associated with COVID-19 in diabetics compared to non-diabetics [8-11].

Among the diabetic patients in our sample, we identified independent associations between T2DM severity (indicated by DCSI score) and both poor COVID-19 clinical outcomes and COVID-19 in-hospital mortality. Many prior studies have investigated the relationship between indicators of diabetes severity, including hemoglobin A1c and fasting plasma glucose, and COVID-19 severity amongst diabetics with inconsistent findings [12,13]. We noted progressively higher odds of both poor COVID-19 clinical outcomes and COVID-19 in-hospital mortality with increasing DCSI scores. To our knowledge, this is the first study to use the DCSI score as an indicator of T2DM severity and to characterize the relationship between DCSI scores and COVID-19 outcomes. DCSI scores are based on ICD-10 codes that can be found within existing EMR data and are therefore relatively straightforward to compute. Our results thus support the development and use of clinical decision support tools utilizing DCSI scores to facilitate decisions regarding treatment aggressiveness and resource allocation for diabetic patients hospitalized with COVID-19.

Many pathophysiological mechanisms for the ways in which diabetes may lead to adverse outcomes associated with COVID-19 have been proposed. Poorly controlled diabetes and the consequent hyperglycemia have been shown to impair the immune response to COVID-19, inhibiting lymphocyte proliferation and weakening immune cells including macrophages and natural killer cells [20-22]. It has also been observed that the chronic inflammatory state and elevated baseline cytokine levels associated with diabetes may augment the intensity of the cytokine response to COVID-19 and increase the likelihood of the cytokine storm that drives multi-organ failure in patients who develop critical COVID-19 illness [21,23,24]. Additionally, patients with diabetes have increased expression of the ACE2 protein that has been shown to be the predominant receptor for the SARS-CoV-2 virus, which may also account in part for the increased susceptibility to severe COVID-19 among diabetics [25,26]. These mechanisms provide some insight into the specific pathways that may have led to the poorer COVID-19 clinical outcomes observed amongst diabetic patients in our sample.

Our study has several limitations. First, all of the individuals we included in our sample were from a single health system, with the vast majority of patients living in Southwest Virginia. Our findings thus likely suffer from issues with external validity and may not be generalizable to other geographies or populations with different sociodemographic compositions. Second, we almost certainly faced some issues with data misclassification and information bias. Although we intentionally selected our exposure variables, outcome variables, and covariates based on the literature and what we expected to be readily available within the EMR, some patients may have experienced outcomes that were not captured in the EMR (e.g., a COVID-19 patient who was discharged from the hospital and later died at home). Furthermore, since this is a real-world retrospective study, it is likely that data for some of our variables were incomplete or inaccurate (e.g., incomplete ICD-10 data on patients who were admitted to our health system for the first time; incomplete COVID-19 vaccination information due to a lack of records in the Virginia Immunization Information System). Finally, we were unable to control for socioeconomic status, access to healthcare, medication use, or specific COVID-19 treatments which may have strengthened our findings.

## Conclusions

In this cohort of individuals hospitalized as a result of COVID-19 in a Southwest Virginia health system, patients with T2DM had a statistically significant increase in the odds of experiencing a poor COVID-19 clinical outcome and in-hospital mortality when compared with non-diabetic individuals. Additionally, among the diabetic patients in our sample, we identified independent associations between T2DM severity (indicated by DCSI score) and both poor COVID-19 clinical outcomes and COVID-19 in-hospital mortality.

Our study adds more nuance to the growing body of evidence regarding the relationship between T2DM and COVID-19 severity. Future directions include probing the predictive value of DCSI scores among ambulatory COVID-19 patients, incorporating data from more diverse settings, and exploring specific pathophysiological mechanisms.

## Appendices

Supplementary methods

DCSI Scores

We scanned the full list of ICD-10 codes available in the EMR for each patient at the time of hospital admission and searched for specific codes associated with retinopathy, nephropathy, neuropathy, cerebrovascular disease, cardiovascular disease, peripheral vascular disease, and metabolic disease based on the ICD-10 translation of the DCSI [18]. We then assigned a subscore of 0, 1, or 2 for each category based on the presence or absence of the relevant ICD-10 codes and summed the seven subscores to compute a single DCSI score for each patient.

Missing Data

Patterns of missing data were explored using Cramér’s V for categorical variables and Cohen’s d for continuous variables [27,28]. The Cramér’s V values for T2DM status, sex, race/ethnicity, current smoking status, vaccination status, poor COVID-19 clinical outcome, and COVID-19 in-hospital mortality were <0.1, and the Cohen’s d values for age and BMI were <0.2, which indicated small effect sizes [29,30]. Based on the calculated Cramér’s V and Cohen’s d values, the data were considered to be missing completely at random (MCAR). As a result, we conducted a complete case analysis, excluding 1,479 patients with missing data on one or more variables. This yielded a final sample size of n = 5,504.
